# The wildland-anthropic interface raster data of the Italy–France maritime cooperation area (Sardinia, Corsica, Tuscany, Liguria, and Provence-Alpes-Côte d'Azur)

**DOI:** 10.1016/j.dib.2021.107355

**Published:** 2021-09-12

**Authors:** Liliana Del Giudice, Bachisio Arca, Carla Scarpa, Grazia Pellizzaro, Pierpaolo Duce, Michele Salis

**Affiliations:** National Research Council of Italy, Institute of BioEconomy (CNR IBE), Sassari, Italy

**Keywords:** Wildland-Urban Interface, Anthropic areas, Mediterranean Basin, Fire risk, GIS, Communities

## Abstract

We applied a geographical information system analysis to reclassify and characterize anthropic buildings based on structure density and area covered, land type, and proximity to wildlands able to originate intense wildfires and spot fires. The methodology was carried out in the 93,000 km^2^ Italy-France Maritime cooperation area (which includes the Regions of Sardinia, Tuscany, and Liguria, in Italy, and Corsica, and Provence-Alpes-Côte d'Azur, in France). We produced a 100-m raster dataset that characterizes and maps medium-high anthropic presence, wildland-anthropic areas, dispersed anthropic areas, and non-anthropic zones, in the whole study area. The study allowed to highlight variations in wildland anthropic interfaces among and within Regions as a function of anthropic presence and types and the surrounding wildlands. The spatial dataset provided with this work represents a valuable contribution to support landscape and urban planning and inform strategies to limit wildfire impacts nearby anthropic areas.

## Specifications Table

**Table:** 

Subject	Forestry; Management, Monitoring, Policy and Law;
Specific subject area	Mapping and characterizing wildland anthropic interfaces
Type of data	Table Figure Geospatial data
How data were acquired	The raw data were extracted from different geospatial files and sources and analysed by GIS software (ArcGIS) tools
Data format	Raster file (*.tif)
Parameters for data collection	Parameters were identified after reviewing existing EU and North America methodologies and approaches related to the topic, taking into account the existing gaps and differences in regional and National databases on vegetation and anthropic area mapping in the study area. We identified and used open-source data
Description of data collection	The geospatial dataset characterizes and maps at fine-scale (100-m resolution) wildland-anthropic interfaces in the 93,000 km2 Italy-France Maritime cooperation area, which includes 3 Regions from Italy (Sardinia, Tuscany and Liguria) and 2 Regions from France (Corsica and PACA). The raster map classifies the study area into the following classes: a) Anthropic (high and medium anthropic presence); b) Wildland-Anthropic (WA Interface and WA Intermix); c) Dispersed Anthropic (DA in Forest Areas, DA in Rural Areas and DA in Non-Vegetated Areas); d) Non-Anthropic (Forest Areas, Rural Areas, Non-Vegetated Areas, Water Bodies
Data source location	10-m Land Cover Map of Europe 2017 (Malinovski et al. 2020), http://s2glc.cbk.waw.pl/extension Open Street Map (OSM) buildings shapefiles, https://www.geofabrik.de/
Data accessibility	With the article

## Value of the Data

• In fire-prone regions, the expansion of the areas where anthropic buildings and wildland vegetation are in contact or intermingled is raising concerns: to mitigate wildfires losses in anthropic zones, basic information and data such as the locations of interface areas or dispersed buildings are required.
• For the whole study area, we derived a 100-m spatial dataset based on four main classes (anthropic, wildland-anthropic interface, dispersed anthropic, non-anthropic) and subclasses using high-resolution input layers. The majority of previous studies on this topic carried out in Europe on large areas focused on peri-urban zones, were based on Corine Land Cover layers, or produced outputs at lower resolutions.
• This study provides a standardized approach to fine-scale characterize and map wildland-anthropic areas in neighbouring areas of the Euro-Mediterranean area, for which a univocal definition of wildland-anthropic interface or intermix is not available due to differences in national and regional legislations.
• Our 100-m Wildland-Anthropic Interface dataset can assist risk monitoring and management activities, particularly in terms of wildfire and flood risk, which are key concerns in the Italy-France Maritime cooperation area. Fuel treatments, prevention and risk-awareness programs, as well as the creation of wildfire adapted communities, can be promoted and optimized considering the specific characteristics and exposure levels of wildland-anthropic areas, from local to provincial or regional scales.
• Fire and land managers, urban planners, and policy makers, may benefit from this geospatial dataset for a number of decision-support activities. Information on wildland anthropic interfaces can be combined with data on socio-economic vulnerability and on wildfire exposure and risk obtained by modelling approaches.

## 1 Data Description

**Fig. 1:**
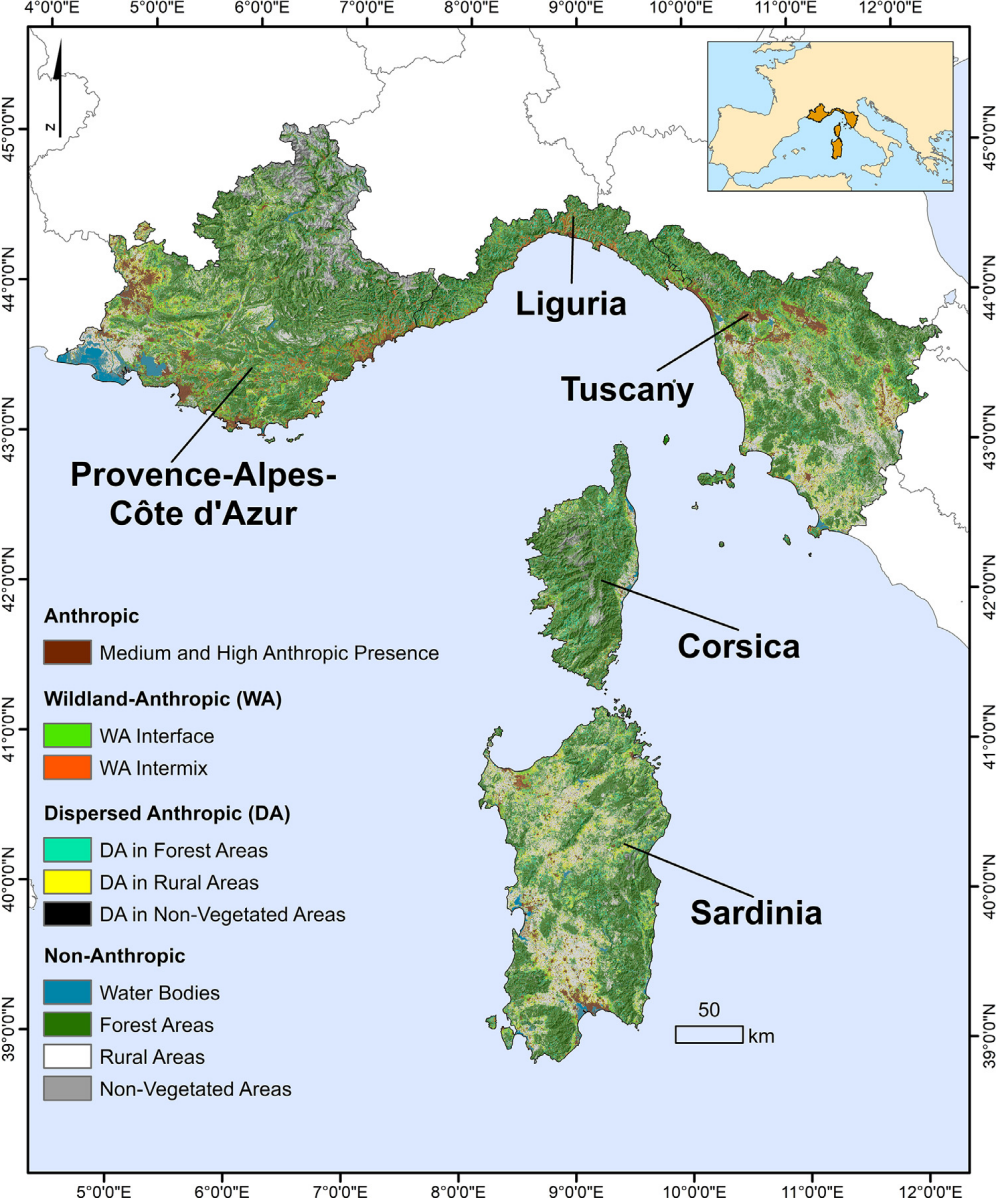
100-m resolution wildland-anthropic interface map of the Italy-France Maritime cooperation area (93,000 km^2^). The study area is classified into 4 main categories (Anthropic, Wildland-Anthropic, Dispersed Anthropic, Non-Anthropic), as a function of anthropic presence or absence, main land cover, and proximity (2-km buffer) to large patches of contiguous forests (> 5 km^2^).

The wildland-anthropic interface map and summary data for the study area are presented in Fig. 1 and in Table 1, respectively, as well as in Supplementary Figures.

**Table 1:** Total area and percentage of the diverse classes of anthropic, wildland-anthropic, dispersed anthropic and non-anthropic areas, considering the whole study area and the five Regions of the Italy-France Maritime cooperation territory. For anthropic, wildland-anthropic and dispersed anthropic classes, the total structure counts (in thousands) are also reported.

	Study Area			Sardinia			Tuscany			Liguria			Corsica			PACA		
	km^2^	%	# str.	km^2^	%	# str.	km^2^	%	# str.	km^2^	%	# str.	km^2^	%	# str.	km^2^	%	# str.
**Anthropic** (Medium and High Anthropic Presence)	4,430.2	4.8	3,000.0	897.1	3.7	265.4	1,346.3	5.9	623.5	180.2	3.3	148.3	70.3	0.8	48.2	1,936.4	6.1	1,914.7

**Wildland-Anthropic (WA)**	9,131.2	9.8	2,373.6	1,098.2	4.6	144.7	2,510.7	10.9	466.2	1,192.8	22.0	298.7	603.0	6.9	181.9	3,726.5	11.7	1,282.1
WA Interface	5,246.0	5.6	1,619.8	792.4	3.3	118.2	1,590.7	6.9	341.9	481.1	8.9	185.6	321.8	3.7	144.7	2,060.0	6.5	829.5
WA Intermix	3,885.3	4.2	753.8	305.9	1.3	26.5	920.0	4.0	124.3	711.7	13.1	113.2	281.2	3.2	37.2	1,666.6	5.2	452.5

**Dispersed Anthropic (DA)**	17,606.3	18.9	474.3	5,602.5	23.2	120.0	4,845.8	21.1	134.9	1,241.7	22.9	38.8	1,221.5	14.0	27.4	4,694.8	14.7	153.2
DA in Forest Areas	8,877.2	9.5	259.7	2,073.8	8.6	83.1	2,406.4	10.5	77.6	1,077.1	19.9	9.1	885.1	10.1	10.0	2,434.8	7.6	79.9
DA in Rural Areas	8,410.7	9.0	203.7	3,432.8	14.2	33.4	2,383.7	10.4	55.5	155.1	2.9	29.3	312.4	3.6	16.2	2,126.7	6.7	69.2
DA in Non-Vegetated Areas	318.3	0.3	10.9	96.0	0.4	3.4	55.6	0.2	1.8	9.4	0.2	0.4	24.0	0.3	1.1	133.3	0.4	4.2

**Non-Anthropic**	61,952.4	66.5		16,535.5	68.5		14,289.3	62.1		2,805.4	51.8		6,833.7	78.3		21,488.6	67.5	
Forest Areas	40,689.2	43.7		9,888.1	41.0		9,324.7	40.6		2,609.1	48.1		5,515.1	63.2		13,352.2	41.9	
Rural Areas	15,827.0	17.0		5,604.7	23.2		4,509.6	19.6		159.7	2.9		738.7	8.5		4,814.5	15.1	
Non-Vegetated Areas	2,948.1	3.2		281.8	1.2		152.4	0.7		10.1	0.2		358.4	4.1		2,145.5	6.7	
Water Bodies	2,488.1	2.7		760.9	3.2		302.7	1.3		26.6	0.5		221.5	2.5		1,176.4	3.7	

**TOTAL**	93,120.2	100	8,695.9	24,133.3	100	794.8	22,992.1	100	1,825.7	5,420.0	100	823.4	8,728.6	100	466.7	31,846.3	100	4,785.3

On the whole, the areas with anthropic presence, which include anthropic, wildland-anthropic, and dispersed anthropic areas, account for about 31,000 km^2^, that is approximately 33% of the study area. Corsica is the Region characterized by the highest values without anthropic areas (about 78.3%), whereas Liguria shows the most significant incidence of anthropic presence (48.2%).

Regarding the medium and high anthropic presence areas, Tuscany and PACA Regions show the highest values, with about 6% of their lands covered by this class. By contrary, Corsica anthropic areas are by far less relevant, with a regional area of about 0.8%. On average, about 4,400 km^2^ of the Italy-France Maritime cooperation territory is covered by areas with medium and high anthropic presence.

Wildland-anthropic (WA) areas represent about 9,100 km^2^ of the whole Italy-France Maritime cooperation area, and include interface and intermix areas, which cover about 5.6% and 4.2% of the study area, respectively. WA interfaces range from a maximum value of 8.9% in Liguria to 3.3% in Sardinia. Overall, Sardinia and Corsica exhibit interface areas lower than 4% of the regional surface, and therefore present much less WA interface than the mainland Regions (above 6.5%). WA intermix areas occupy large zones of Liguria (13.1% of the regional area), while the other Regions present lower percentages, with the minimum of about 1.3% in Sardinia.

About 17,600 km^2^ of the study area is characterized by dispersed anthropic buildings: the most of dispersed anthropic areas is concentrated in forest (9.5%) and rural (9.0%) landscapes. Dispersed buildings located in forest zones are substantially high in Liguria (19.9% of the regional area), which basically doubles the values of the other Regions (minimum value of dispersed anthropic buildings in forest areas is shown by PACA, with about 7.6%). As far as dispersed buildings in rural landscapes are concerned, Sardinia and Tuscany exhibit the highest percentage, with about 14.2% and 10.4% of their regional area, respectively. Conversely, Corsica and Liguria show low presence of dispersed anthropic in rural zones, with values below 3.6%.

The areas characterized by absence of anthropic buildings are largely represented by forest landscapes, which account for about 40,700 km^2^, that is 43.7% of the Italy-France Maritime cooperation area. All Regions present forest areas without anthropic presence above 40%, with the peak observed in Corsica (63.2%). Rural areas without anthropic buildings cover on average 17.0% of the study area, even if with high interregional variations (from 23.2% in Sardinia to 2.9% in Liguria).

## 2 Experimental Design, Materials and Methods

### 2.1 Study area

The study area encompasses about 93,000 km^2^ of land in the Euro-Mediterranean region, between 39°00’–45°00’ N latitude and 5°00’–12°00’ E longitude. It covers the Italy-France Maritime cooperation area, which includes three Italian regions, namely Sardinia, Tuscany and Liguria, and two French regions, Corse and Provence-Alpes-Côte d'Azur (PACA). About 12 million people live in the area, even if this number grows in spring and summer due to the touristic fluxes towards highly-valued landscapes, coastal and cultural areas. Wildfires represent the most relevant threat affecting forests and rural zones, natural resources and anthropic values of the study area: in the period 2002–2016, about 120,000 wildfire ignitions were observed in the study area, with a total area burned close to 405,000 ha.

### 2.2 Anthropic areas and land use data

We derived anthropic area maps and data of the Italy-France Maritime cooperation area from Open Street Map (OSM) buildings shapefiles available in [1]. The OSM buildings are manually digitized as an area along the building outline using an editor supporting geo-rectified aerial imagery as a background. Each structure is represented with its footprints as a closed way or a multi-polygon relation. OSM features are mapped from public domain high-resolution imagery sources, with resolution ranging from 30 to 60 cm. An accuracy analysis of this layer for the study area was not carried out.

To characterize fine-scale land uses, we used the 10-m Land Cover Map of Europe 2017 [2]. These raster data were a product of the S2GLC project and were obtained by a classification of over 15,000 Sentinel-2 images based on algorithms and software, as described in [2]. The legend of the Land Cover Map of Europe 2017 consists of 13 land cover classes. The accuracy assessment of the 10-m Land Cover Map of Europe 2017 revealed high thematic overall accuracy (86.1%) on a continental scale, and average overall accuracy of 86.5% at country level [2].

### 2.3 Geospatial analysis

Reference approach and values to map wildland-urban interfaces were obtained from previous studies carried out in conterminous US [3] and in Catalonia (Spain) [4]. To produce our raster dataset, we adapted some methodological steps of the above works, as described below.

We clipped the OSM buildings shapefile by the five Regions of the Italy-France Maritime cooperation area and we obtained a 2.2 Gb shapefile. We derived location centroids of all anthropic blocks (about 6 million) of the study area, and we generated a point shapefile. We then created a 100-m raster file with anthropic buildings density (normalized in # centroids / km^−2^), using as reference a 300-m gridded shapefile. The pixels containing anthropic buildings were reclassified as: no buildings (no buildings km^−2^), low (< 75 centroids km^−2^), medium (75–250 centroids km^−2^) and high (≥ 250 centroids km^−2^) density areas. In addition, we calculated the area covered by each building block and then quantified the overall and the percent area covered by anthropic structures at 100-m resolution, using as reference the 300-m gridded shapefile. Depending on the percentage of area covered by anthropic blocks, the pixels with buildings were reclassified into four classes (0; 0–1%; 1–10%; ≥10%). Pixels without anthropic buildings, according to both centroid locations and area covered by buildings, were classified as Non-Anthropic Areas.

As a second step, we clipped the 10-m Land Cover Map of Europe 2017 by the study area, and we derived a 2.0 Gb raster file. The original land use classes of the dataset were reclassified into the following main types: Forest; Rural; Non-Vegetated; Water Bodies. The classification scheme used to derive the above main land cover types from the Land Cover Map of Europe 2017 is reported in Table 2. We then resampled at 100-m resolution the reclassified raster data using the Majority Resampling Technique of ArcMap 10.8.

**Table 2:** Classification scheme used to derive the four main land cover types of the study area, starting from the classes of the Land Cover Map of Europe 2017 [2].

Land Cover Map of Europe 2017 Classes	Main Land Cover Types
Broadleaf tree cover	Forest
Coniferous tree cover	Forest
Moors and Heathland	Forest
Sclerophyllous vegetation	Forest
Cultivated areas	Rural
Vineyards	Rural
Herbaceous vegetation	Rural
Peatbogs	Rural
Artificial surfaces and constructions	Non-Vegetated
Natural material surfaces	Non-Vegetated
Permanent snow covered surfaces	Non-Vegetated
Marshes	Water Bodies
Water bodies	Water Bodies

We then created the intense wildfire and ember exposure grids, which represent the areas where the risk of fire ignitions from embers originated by dense canopy fuels, as well as the risk of occurrence of high-intensity wildfires due to high fuel loads, is significant. For this purpose, we identified the dense forest areas by first combining the 10-m Land Use Cover of Europe with a 100-m gridded shapefile, and then by assigning the code “dense forest” only to those pixels characterized by a percentage of forest classes higher than 50%, spatially contiguous and covering areas larger than 5 km^2^. We then detected the areas that may be exposed to ember showers and high-intensity events by a 2-km buffer from dense forest areas.

**Fig. 2:**
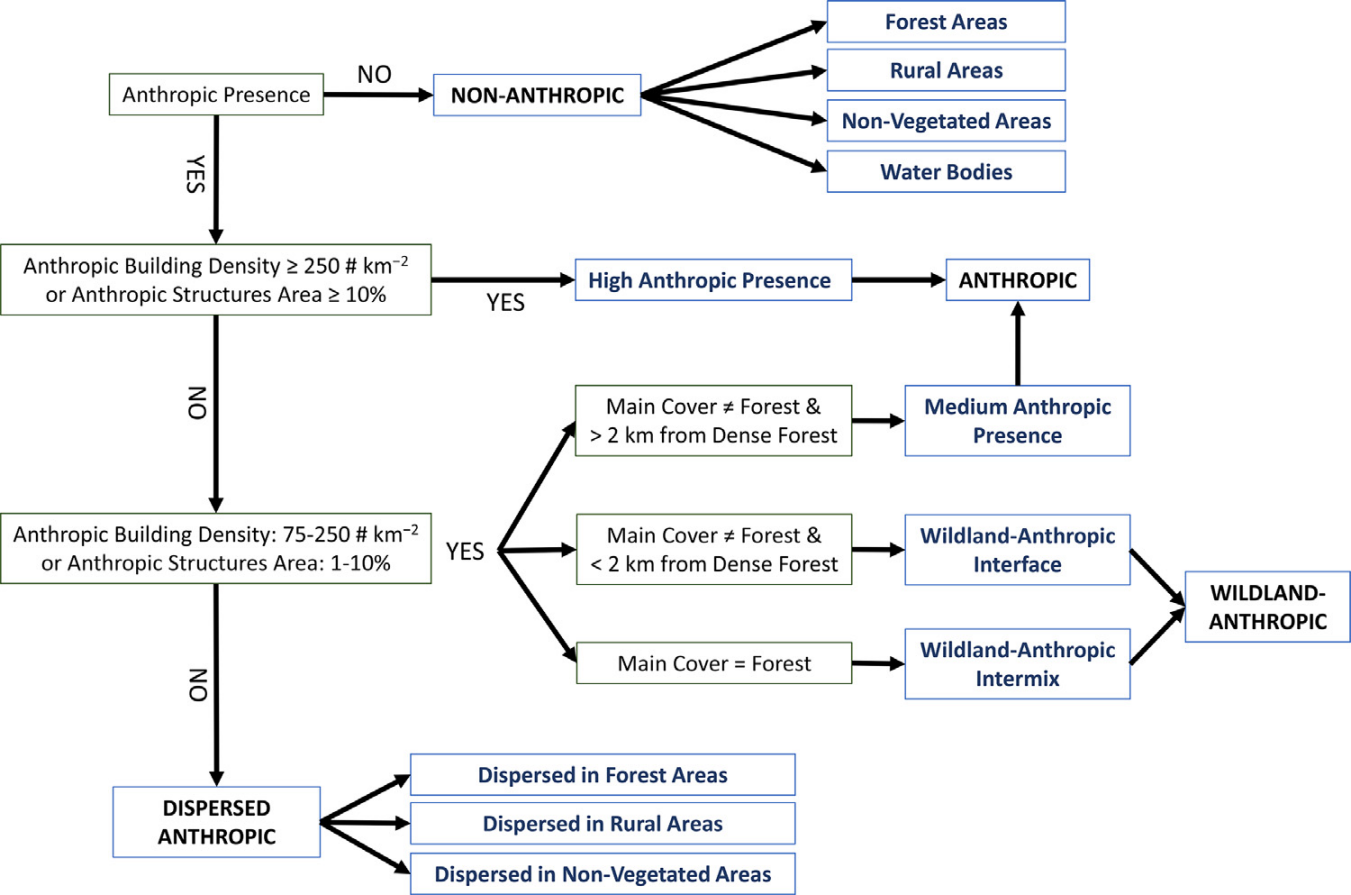
Flowchart of the methodology used in this work to identify and map Anthropic, Wildland-Anthropic, Dispersed Anthropic and Non-Anthropic areas. The above 4 classes were identified as a function of anthropic presence (area covered by anthropic blocks and anthropic building density), main cover type, and distance (2-km buffer) from dense and contiguous wildlands (> 5 km^2^), as described in the Methods.

Combining the abovementioned raster data at 100-m resolution (anthropic building density; percentage of area covered by anthropic blocks; main land cover types; intense wildfire and embers exposure grid) we derived the wildland-anthropic interface map of the study area, which was based on the methodology summarized in Fig. 2.

We obtained the following classes:

a) *Anthropic*: (1) high anthropic presence (anthropic buildings density ≥ 250 buildings km^−2^ or percentage of anthropic structures ≥ 10%); (2) medium anthropic presence (main cover ≠ forest, anthropic buildings density 75–250 buildings km^−2^ or percentage of anthropic structures 1–10%, and buildings located > 2 km from dense forests);
b) *Wildland-Anthropic*: (1) WA Interface (main cover ≠ forest, anthropic buildings density 75–250 buildings km^−2^ or percentage of anthropic structures 1–10%, buildings located < 2 km from dense forests); (2) WA Intermix (main cover = forest, anthropic buildings density 75–250 buildings km^−2^ or percentage of anthropic structures 1–10%);
c) *Dispersed Anthropic*: (1) DA in Forest Areas (main cover = forest, anthropic buildings density < 75 buildings km^−2^ or percentage of anthropic structures < 1%); (2) DA in Rural Areas (main cover = rural, anthropic buildings density < 75 buildings km^−2^ or percentage of anthropic structures < 1%); (3) DA in Non-Vegetated Areas (main cover = non-vegetated, anthropic buildings density < 75 buildings km^−2^ or percentage of anthropic structures < 1%)
d) *Non-Anthropic*: (1) Forest Areas (main cover = forest, absence of anthropic buildings); (2) Rural Areas (main cover = rural, absence of anthropic buildings); (3) Non-Vegetated Areas (main cover = non-vegetated, absence of anthropic buildings) (4) Water Bodies (main cover = water bodies, absence of anthropic buildings).

## Ethics Statement

Hereby, the authors of this work consciously assure that for this manuscript the following is fulfilled:

1) The manuscript adheres to Ethics in publishing standards.
2) This material is the authors' own original work, which has not been previously published elsewhere.
3) The paper is not currently being considered for publication elsewhere.
4) The paper reflects the authors' own research and analysis in a truthful and complete manner.
5) The paper properly credits the meaningful contributions of each author; all authors have been personally and actively involved in substantial work leading to the paper, and will take public responsibility for its content.
6) The results are appropriately placed in the context of prior and existing research.
7) All sources used are properly disclosed.
8) Our work does not involve human subjects, animal experiments or data collected from social media platforms.

## CRediT Author Statement

**Liliana Del Giudice:** Writing, Methodology, Software; **Bachisio Arca:** Software, Validation; **Carla Scarpa:** Investigation; **Grazia Pellizzaro:** Validation; **Pierpaolo Duce:** Funding acquisition, Conceptualization; **Michele Salis:** Writing, Methodology, Conceptualization, Supervision.

## Declaration of Competing Interest

The authors declare that they have no known competing financial interests or personal relationships which have or could be perceived to have influenced the work reported in this article.

## Appendix Supplementary materials

Image, application 1

Image, image 2

Image, image 3

Image, application 4

Image, application 5

Image, application 6

Image, application 7

Image, application 8

Image, application 9

## References

[1] The Openstreetmap data files, Europe. https://www.geofabrik.de/, 2021 (accessed 02 July 2021).

[2] Malinowski R., Lewiński S., Rybicki M., Gromny E., Jenerowicz M., Krupiński M., Nowakowski A., Wojtkowski C., Krupiński M., Krätzschmar E., Schauer P. (2020). Automated Production of a Land Cover/Use Map of Europe Based on Sentinel-2 Imagery. Remote Sens..

[3] S. Martinuzzi, S.I. Stewart, D.P. Helmers, M.H. Mockrin, R.B. Hammer, V.C. Radeloff, The 2010 wildland-urban interface of the conterminous United States, Research Map NRS-8. Newtown Square, PA: US Department of Agriculture, Forest Service, Northern Research Station. 124 p., 8 (2015): 1-124

[4] Alcasena F.J., Evers C.R., Vega-Garcia C. (2018). The wildland-urban interface raster dataset of Catalonia. Data Br..

