# Corrigendum to “The wildland-anthropic interface raster data of the Italy–France maritime cooperation area (Sardinia, Corsica, Tuscany, Liguria, and Provence-Alpes-Côte d'Azur)” [Data in Brief Volume 38 (2021) Article 107355]

**DOI:** 10.1016/j.dib.2023.109023

**Published:** 2023-02-24

**Authors:** Liliana Del Giudice, Bachisio Arca, Carla Scarpa, Grazia Pellizzaro, Pierpaolo Duce, Michele Salis

**Affiliations:** National Research Council of Italy, Institute of BioEconomy (CNR IBE), Sassari, Italy

The authors regret to declare that a Table that appears in the published article (Table 1) presented a few mistakes. In the amended table, the values in the final row of the “TOTAL” columns are calculated considering the subtotals of each class category, and not considering the whole set of values. The correct version of the table is attached in this corrigendum.

**Table 1:** Total area and percentage of the diverse classes of anthropic, wildland-anthropic, dispersed anthropic and non-anthropic areas, considering the whole study area and the five Regions of the Italy-France Maritime cooperation territory. For anthropic, wildland-anthropic and dispersed anthropic classes, the total structure counts (in thousands) are also reported

	Study Area			Sardinia			Tuscany			Liguria			Corsica			PACA		
	km^2^	%	# str.	km^2^	%	# str.	km^2^	%	# str.	km^2^	%	# str.	km^2^	%	# str.	km^2^	%	# str.
**Anthropic** (Medium and High Anthropic Presence)	4,430.2	4.8	3,000.0	897.1	3.7	265.4	1,346.3	5.9	623.5	180.2	3.3	148.3	70.3	0.8	48.2	1,936.4	6.1	1,914.7

**Wildland-Anthropic (WA)**	9,131.2	9.8	2,373.6	1,098.2	4.6	144.7	2,510.7	10.9	466.2	1,192.8	22.0	298.7	603.0	6.9	181.9	3,726.5	11.7	1,282.1
WA Interface	5,246.0	5.6	1,619.8	792.4	3.3	118.2	1,590.7	6.9	341.9	481.1	8.9	185.6	321.8	3.7	144.7	2,060.0	6.5	829.5
WA Intermix	3,885.3	4.2	753.8	305.9	1.3	26.5	920.0	4.0	124.3	711.7	13.1	113.2	281.2	3.2	37.2	1,666.6	5.2	452.5

**Dispersed Anthropic (DA)**	17,606.3	18.9	474.3	5,602.5	23.2	120.0	4,845.8	21.1	134.9	1,241.7	22.9	38.8	1,221.5	14.0	27.4	4,694.8	14.7	153.2
DA in Forest Areas	8,877.2	9.5	259.7	2,073.8	8.6	83.1	2,406.4	10.5	77.6	1,077.1	19.9	9.1	885.1	10.1	10.0	2,434.8	7.6	79.9
DA in Rural Areas	8,410.7	9.0	203.7	3,432.8	14.2	33.4	2,383.7	10.4	55.5	155.1	2.9	29.3	312.4	3.6	16.2	2,126.7	6.7	69.2
DA in Non-Vegetated Areas	318.3	0.3	10.9	96.0	0.4	3.4	55.6	0.2	1.8	9.4	0.2	0.4	24.0	0.3	1.1	133.3	0.4	4.2

**Non-Anthropic**	61,952.4	66.5		16,535.5	68.5		14,289.3	62.1		2,805.4	51.8		6,833.7	78.3		21,488.6	67.5	
Forest Areas	40,689.2	43.7		9,888.1	41.0		9,324.7	40.6		2,609.1	48.1		5,515.1	63.2		13,352.2	41.9	
Rural Areas	15,827.0	17.0		5,604.7	23.2		4,509.6	19.6		159.7	2.9		738.7	8.5		4,814.5	15.1	
Non-Vegetated Areas	2,948.1	3.2		281.8	1.2		152.4	0.7		10.1	0.2		358.4	4.1		2,145.5	6.7	
Water Bodies	2,488.1	2.7		760.9	3.2		302.7	1.3		26.6	0.5		221.5	2.5		1,176.4	3.7	

**TOTAL**	93,120.2	100	5,847.9	24,133.3	100	530.1	22,992.1	100	1,224.6	5,420.0	100	485.8	8,728.6	100	257.4	31,846.3	100	3,350.0

The authors apologize for any inconvenience caused.

