# Supplementary material for: The wildland-anthropic interface raster data of the Italy–France maritime cooperation area (Sardinia, Corsica, Tuscany, Liguria, and Provence-Alpes-Côte d'Azur)
Source: Data Brief. 2021 Sep 12;38:107355. doi: 10.1016/j.dib.2021.107355 (PMC8449076; doi:10.1016/j.dib.2021.107355)
Supplement: Supplementary file 1 [file mmc1.docx]

Supplementary Figure 1. Wildland-anthropic interface map of the islands of Sardinia (Italy) and Corsica (France).

Supplementary Figure 2. Wildland-anthropic interface map of Tuscany (Italy), Liguria (Italy) and PACA (France) Regions.
