# Supplementary figures and images for: The wildland-anthropic interface raster data of the Italy–France maritime cooperation area (Sardinia, Corsica, Tuscany, Liguria, and Provence-Alpes-Côte d'Azur)
Source: Data Brief. 2021 Sep 12;38:107355. doi: 10.1016/j.dib.2021.107355 (PMC8449076; doi:10.1016/j.dib.2021.107355)

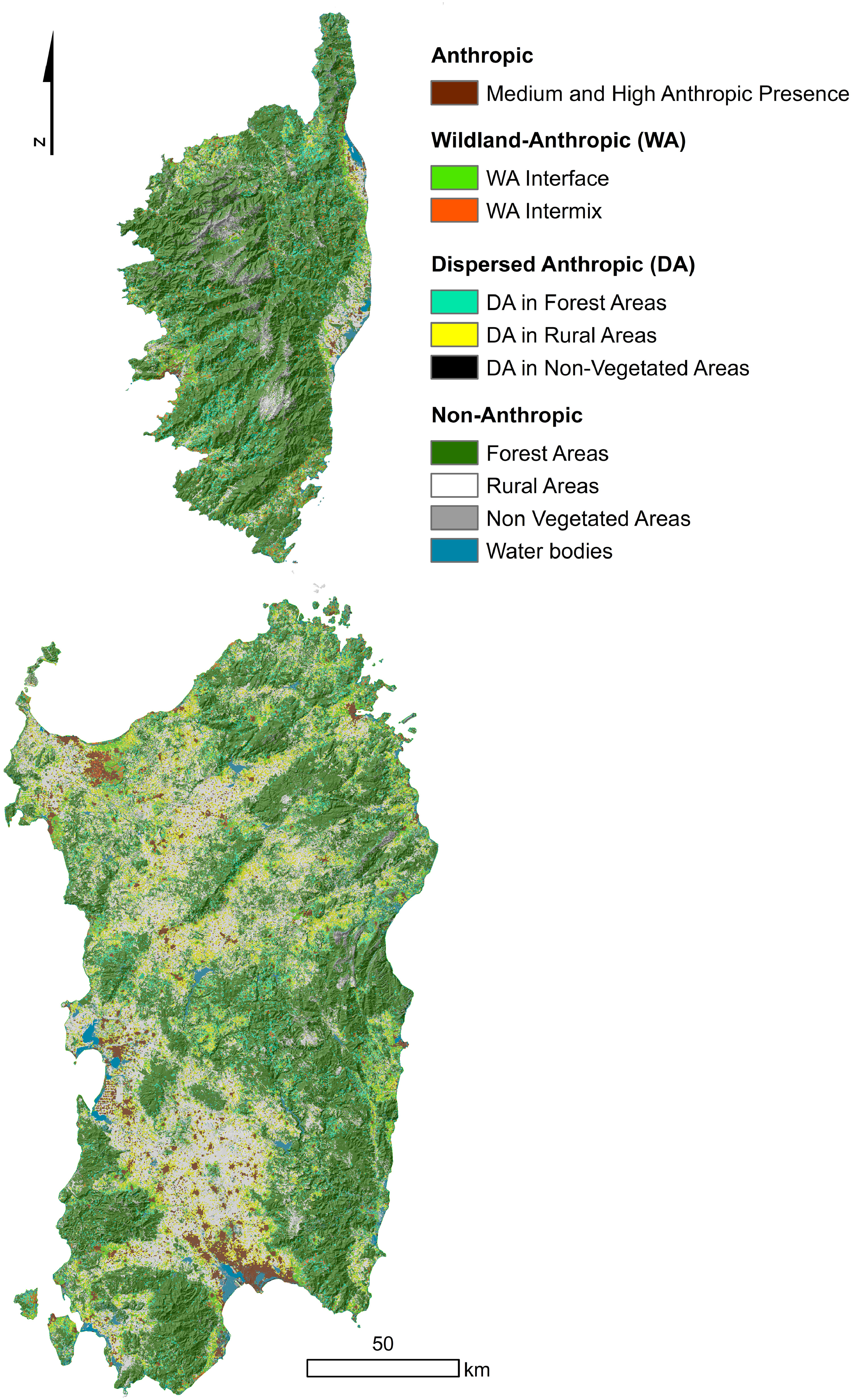

Supplement: Supplementary file 2 [file mmc2.jpg]

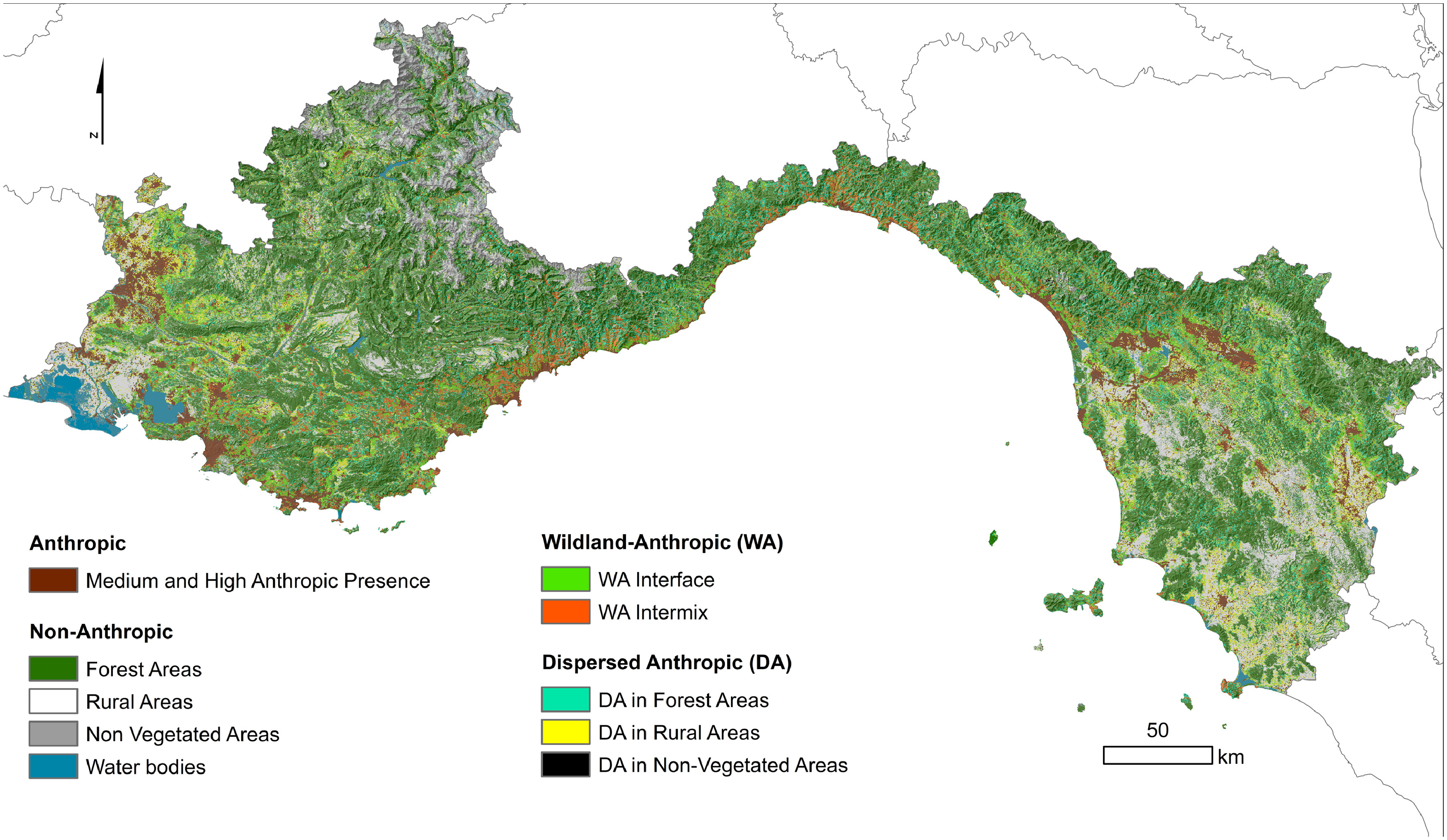

Supplement: Supplementary file 3 [file mmc3.jpg]

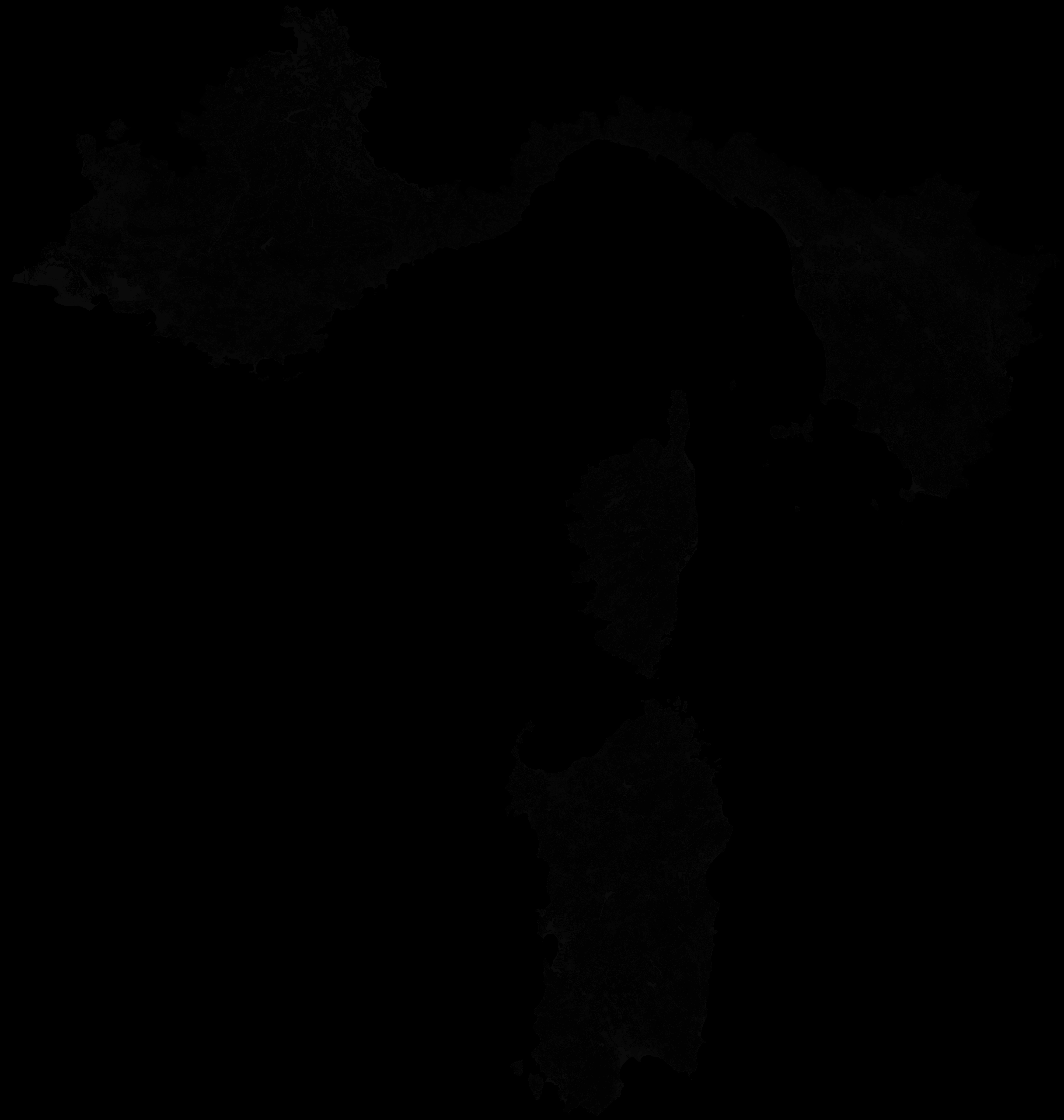

Supplement: Supplementary file 6 [file mmc6.zip › mmc6.tif]

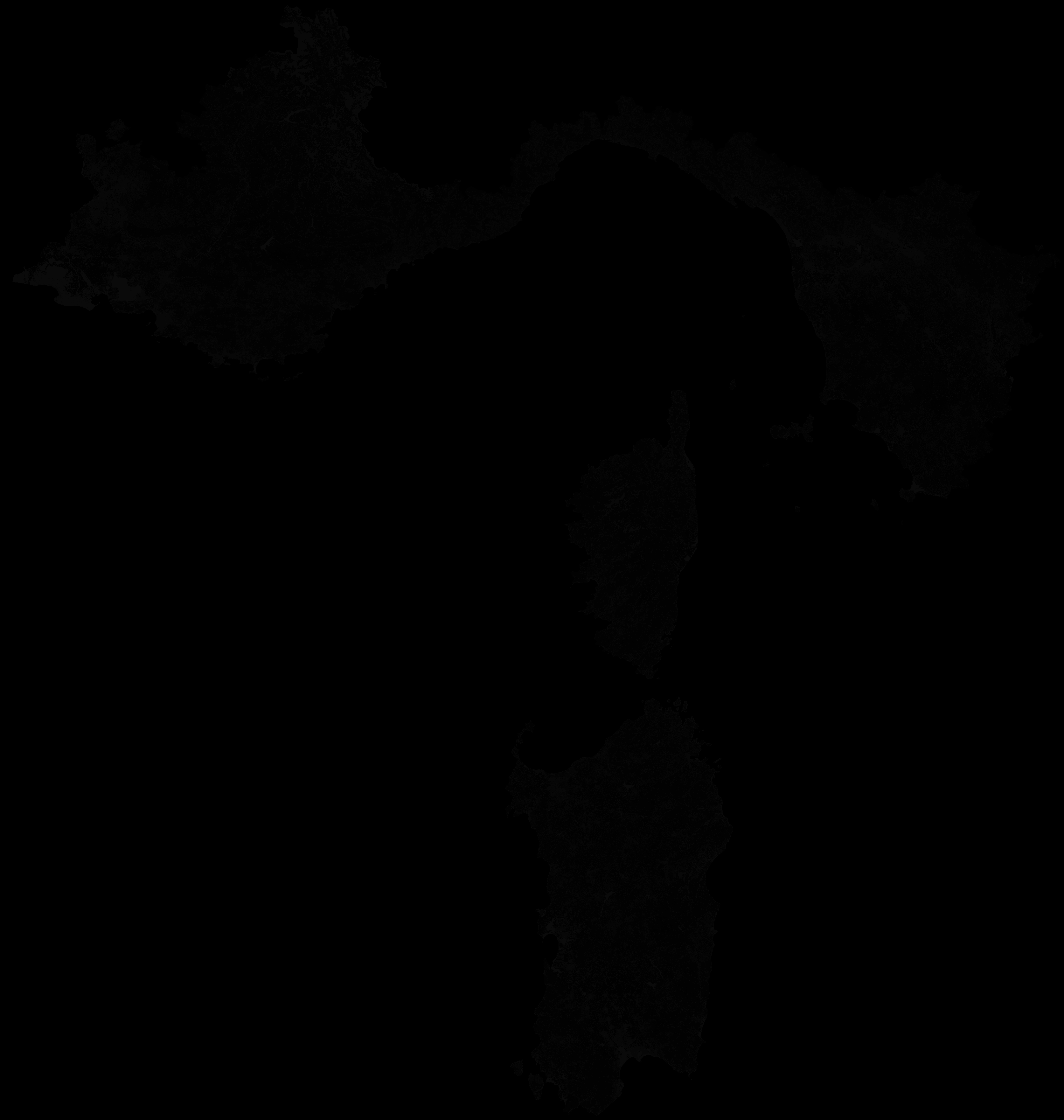

Supplement: Supplementary file 8 [file mmc8.zip › mmc8.ovr]
